# The new cultural norm: reasons why UK foundation doctors are choosing not to go straight into speciality training

**DOI:** 10.1186/s12909-020-02157-7

**Published:** 2020-08-27

**Authors:** Alexander Conor Hollis, Jack Streeter, Clare Van Hamel, Louise Milburn, Hugh Alberti

**Affiliations:** 1 UK Foundation Programme Office St Chad’s Court, 213 Hagley Road, Edgbaston, Birmingham, B16 9RG UK; 2 Address: Flat 305, 89 Branston Street, Birmingham, B186BU UK; 3 School of Medical Education, Cookson Building, Newcastle University, Medical School, Framlington Place, Newcastle upon Tyne, NE2 4HH UK

**Keywords:** Speciality training, Junior doctors, Foundation doctors

## Abstract

**Background:**

The number of UK foundation doctors choosing to go straight into speciality training has fallen drastically over the last 10 years: We sought to explore and understand the reasons for this change.

**Methods:**

We undertook semi-structured interviews with 16 foundation year two doctors, who had not applied to speciality training, from two regional foundation schools. Transcripts were thematically analysed.

**Results:**

The reasons that foundation doctors are choosing not to go straight into speciality training centre around the themes of feeling undervalued, career uncertainty and a new cultural norm. They report major feelings of uncertainty regarding career choice at such an early stage of their profession and this challenge was magnified by a perceived lack of flexibility of training and the growing normality of taking time out from training. Trainees feel a lack of support in planning and undertaking an “FY3” year and being helped back into the workforce.

Trainees overwhelmingly reported that they feel undervalued by their employers. Importantly, however, not going into training directly was not always a reflection of dissatisfaction with training. Many trainees spoke very positively about their planned activities and often saw a break in training as an excellent way to recharge, develop skills and prepare for the rest of their careers in medicine.

**Conclusions:**

Taking a year or more out of training after foundation years has become the new cultural norm for UK junior doctors and reasons for this include feeling undervalued, career uncertainty and the perception that this is now “normal”. Exploring these factors with participants has generated a number of recommendations related to improving the workplace environment, allowing more flexibility in training and supporting those who chose to take an FY3.

## Summary box

Section 1: What is already known on this topic

- The number of junior doctors choosing to enter speciality training directly is has been steadily declining since 2011.
- Instead, junior doctors are increasingly choosing to take time out following completion of the foundation programme and this year has become unofficially known as an ‘FY3 year’.
- It is unclear what factors are driving this phenomenon and we need to better understand trainees needs at this crucial point of their career both to better support them and to allow for effective workforce planning.

Section 2: What this study adds.

- Our study suggests that the increase in trainees deciding to take FY3 years is a result
- of a combination of trainees feeling undervalued by their employers, growing careers uncertainty and changing cultural norms within the cohort.
- Trainees are responding to a perceived lack of flexibility in speciality training and taking time out as a means of regaining control and autonomy over their working and personal life.

## Background

The numbers of UK foundation doctors deciding to move directly to specialty training has been declining since 2011 with only 37.7% entering specialty training in 2018 [1]. This is not a novel issue and concerns over this phenomenon have been present since 2010 when ‘only’ 70% of doctors were entering training directly [2]. Arguably more worrying for the profession, the numbers opting for a break from working as a doctor altogether following foundation training has also risen sharply to a high of 14.4% of trainees in 2018 [3].

Until recently there has been little research exploring the reasons behind these observed developments, although the GMC [4] and the BMA [5] amongst other researchers [6, 7] have produced some preliminary work. The reasons postulated to explain this trend have been cited as trainee burnout, poor rotas, an increasing desire for improved work life balance, system pressures or a combination of these. A novel discrete choice experiment studied revealed that trainees place most value in their training post on good working conditions, good opportunities for their partners and desirable geographical location [8]. More recent work has suggested some differences in socio-economic status between trainees who do and do not take time out of the training pathway [9]. A lack of flexibility may also be key with some experts describing the current system as “an inflexible training pipeline” [10].

However, the majority of the data collected has been from quantitative surveys and has not been able to explore these reasons in detail. Focus groups have been used to contribute to both the national BMA and GMC reports [5, 11], but the research has focused on collating lists of reasons why doctors are leaving rather than exploring these reasons in depth from a trainee perspective.

In the UK, after completing their undergraduate studies, doctors undertake 2 years of foundation training in which they rotate around six different specialties. Following this, they can apply immediately or later to a specialty training programme which lasts between three to 7 years depending on their chosen area. This will take them through to the completion of their training as a hospital consultant of General Practitioner. Therefore, any widening of an interruption in this conveyer belt will have negative repercussions on senior doctor recruitment in the future.

Historically the prevailing narrative both in the medical community and media has been that the growing exodus of junior doctors taking a year or more out after foundation training, often called an FY3 year, is in order to travel or work abroad [7]. However, the latest data from the Foundation Programme Careers Destination Survey 2018 [1] shows that the percentage choosing to work abroad has actually remained relatively static from 2011 (11.9%) to 2018 (11.3%). It is in fact those choosing to take a service post in the UK (2.3% in 2011 to 17.6% in 2018) and those choosing to take a career break (4.7% in 2011 to 14.8% in 2018) that have seen the most significant increase. Much of the current research focuses on where FY2 doctors are going and not *why* they are choosing not to enter specialty training. This is a dynamic and rapidly evolving topic which requires further exploration. Illuminating the reasons behind the trend in increasing career breaks and investigating potential actions that could alleviate these factors for trainees would inform policy makers as they make workforce plans for the future.

## Methods

### Participant recruitment

Recruitment was from two contrasting Health Education regions: the Northern Foundation School has a low proportion of trainees entering speciality training (37.1%, *n* = 388, 2018), in contrast with the West Midlands (North, South and Central combined) Foundation School which has a relatively high proportion of trainees entering speciality training (45.4%, *n* = 504, 2018). The range across regions varied from 30 to 53% [1].

All Foundation Year two (FY2) doctors in the two regions were invited by email to participate if they had not applied to start speciality training directly after their FY2 year. Graduates of medical schools in the United Kingdom are required to work for 2 years as foundation doctors prior to entering speciality training as a GP or hospital-based specialist. They were invited to email the research team if interested, who then contacted the volunteers to arrange an appropriate time for the interview. Once data saturation was reached no further volunteers were contacted. Interviews took place during the participants final 6 months of their FY2 year.

### Semi structured interviews

Semi structured interviews were conducted either over the phone or online via video link. Prospective participants were given information about the study and the interview and informed written consent was taken. The interviews were conducted by two junior doctors, one FY2 (AH) and one GPST3 (JS). The interview schedule was developed from the literature on the topic and based on exploring the participants reasons for not going straight into speciality training.

### Ethical considerations

No identifiable details or participants names were stored with the interviews. Each interview was given a code and was recorded securely on a recording device. Transcription was undertaken verbatim by a professional service. Audio files were deleted following transcription. Ethical approval was granted by Newcastle University.

### Data analysis

Thematic analysis was undertaken using the Braun and Clarke method [12]; this involved data familiarisation, generating initial codes, and reviewing and defining themes. JS and AH coded the transcripts initially and a proportion of transcripts were second coded by CH and HA. The manuscripts from the 16 interviews were coded to identify themes and sub-themes; data saturation appeared to have been reached at this point. Data analysis was undertaken iteratively. The final model emerged following discussions (by teleconference and face-to-face) with all team members. Once this final model was generated, we reviewed the themes and manuscripts again and had a final verification via teleconference meeting with all researchers involved in coding AH, CH, CVH and HA.

### Researcher characteristics and reflexivity

AH was a foundation doctor at the time of this study, JS and JM were both GP trainees. CVH and HA have both achieved CCT in Anaesthetics and GP respectively. There were many potential influences between the researchers own characteristics and the research question and participants. However, as the researchers were from a range of backgrounds and at different stages of training this prevented a single perspective predominating and dominating the study.

## Results

Interview manuscripts were analysed using a coding framework and three broad themes emerged which centred around the aim of exploring reasons why foundation doctors are not directly entering training. In addition, a fourth theme emerged from the data, related to participants perceptions of the FY3 year, including both issues that concerned them and suggestions for improvement generated.

Participants were prospectively interviewed and therefore often did not have set plans for their FY3 year. Table 1 above shows that most participants had plans which included several options; travel and locum work being the most popular options amongst their plans.

**Table 1:**
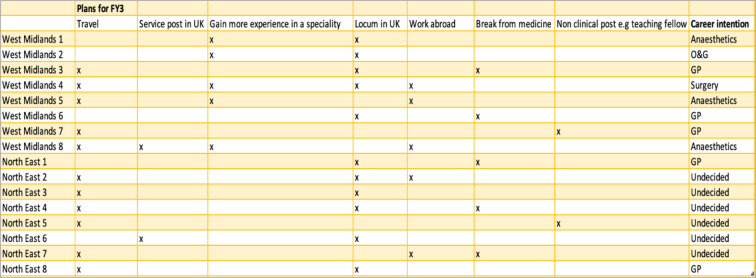
Participants prospective plans for FY3 year

**Fig. 1:**
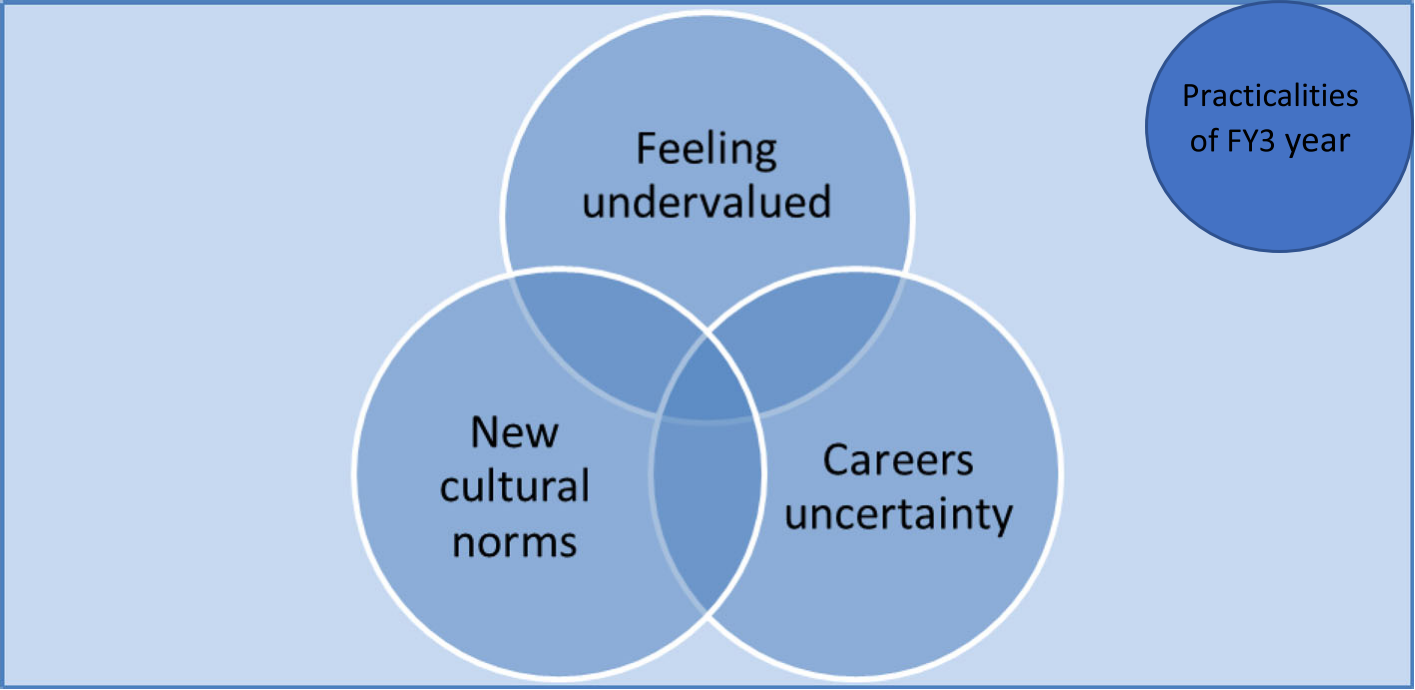
Conceptual model of the central themes which emerged from the data

The three central themes which emerged from the data had interlinking data and significant overlap and are presented in Fig. 1.

**Fig. 2:**
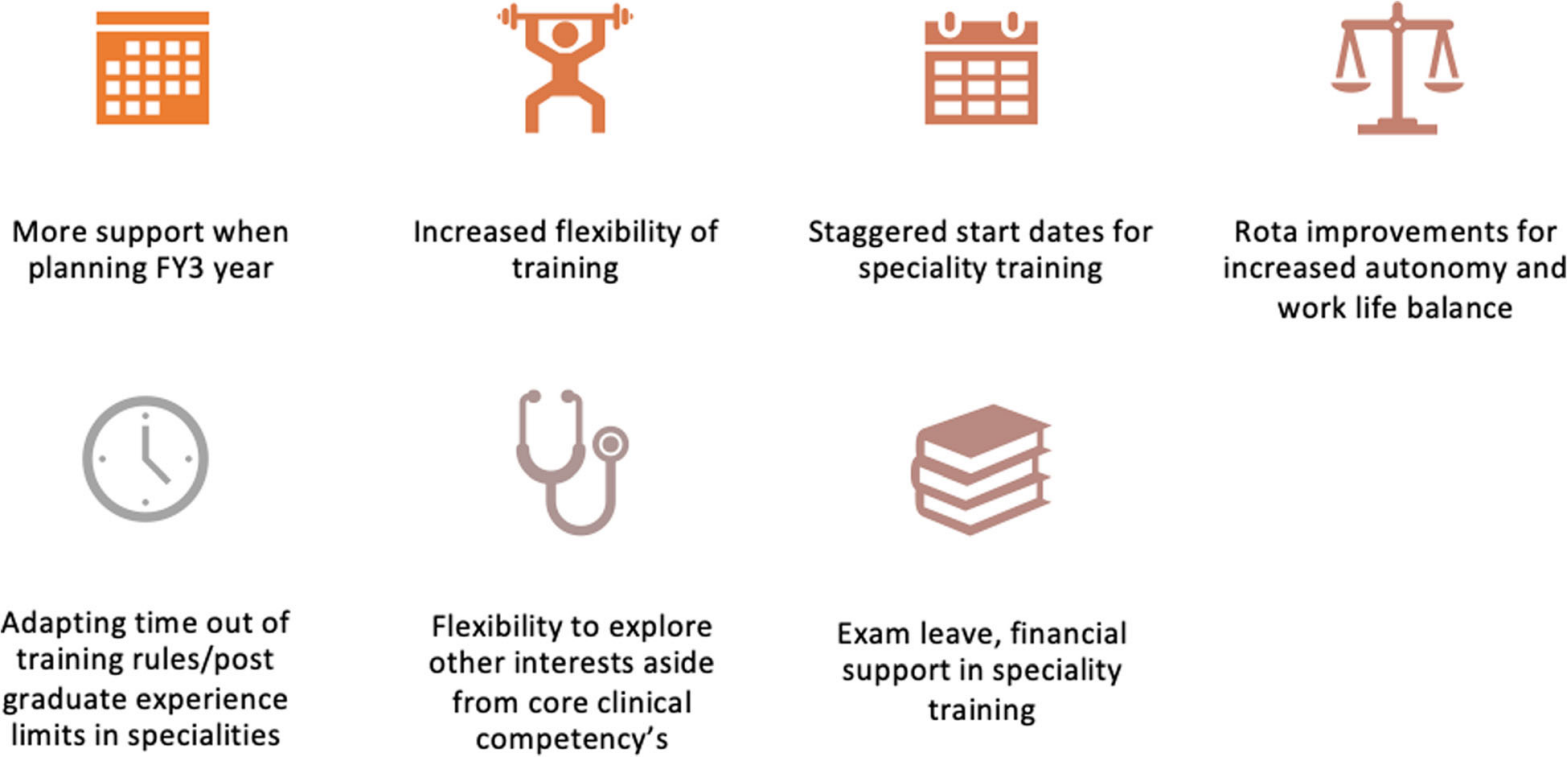
Suggestions for improvement to foundation training

### Theme one: feeling undervalued – ‘you feel like you are doing two people’s job’

Foundation doctors expressed strong feelings of feeling undervalued, often citing lack of flexibility within their roles and rotas. Participants talked about feeling that they were only valued for service provision, using examples of being unable to secure annual leave to attend significant family events or take holiday. The view that they were the ‘cheapest option’ to fill rota holes left doctors feeling that they felt undervalued as ‘you are doing two peoples jobs and also kind of undervalued because of that’. The suggestion amongst those interviewed was clearly that the balance has moved away from being trained for a future career and more towards being responsible for mundane jobs which held little benefit for personal development.

*“especially in my FY1 a lot of the tasks I did almost felt administrative and I wasn’t learning or using what I’d learnt throughout medical school.”*

The facilities for doctors within the trusts was a strong sub-theme within the data, with doctors commenting that poor rest facilities, limited access to catering facilities and expensive, limited parking impacted on their feelings of being unappreciated. This negatively impacted on doctor’s feelings of self-worth. Doctors felt that the pressure put on them in the workplace from workload, pressure to cover rota gaps and poor facilities increased their frustration at work and negatively impacted on their feelings of being appreciated.

*“that’s just a completely crap atmosphere to be working in never mind training”.*

Participants raised issues with poor workplace administration, saying ‘you know nothing is going to be straight forward, you are going to have to chase people around’ in relation to getting timetables, rotas and difficulties with requesting annual leave, being paid correctly and claiming expenses. This meant that even those who felt motivated to request study leave or try to work towards outside goals were frustrated by the administration processes required for these and sometimes unable to achieve these goals because of this:

*“the hope of having flexibility in a locum year is that I could take a couple of weeks of to get you know, some revision done”.*

### Theme two: career uncertainty – ‘you’ve got to make sure you go for what you actually want to do’

The majority of those interviewed expressed some uncertainty about their career choices. The common theme was that it was ‘too early’ to decide on a specific career path, magnified by the lack of flexibility within training schemes and the difficulties faced if you want to change between speciality.

*“You’ve done half an F2 job when you have to apply, I think if there was more support and signposting through F1 then I think that would be better”.*

Those who had not had experience of the careers they were applying to mentioned that the lack of flexibility within foundation training and difficulty securing ‘taster weeks’ where you can experience a week within the specialty you are considering impacted on their ability to commit to a pathway of training.

*“I’m committing myself to another eight years of training and you don’t want to get that wrong”.*

A strong theme within the data was the feeling that if you did not want to take time out of training you would be disadvantaged when compared to those who did, and specifically those who had used that time to experience specialties they were considering and had improved their CV with reference to specialty training. This also crosses over significantly with the third theme, a new culture within training.

*“So I had also considered applying now but deferring for a year. But I just felt overall partly because of time constraint like I just didn’t, by the time I’d had those conversations you know it was a few weeks until applications were due and I just didn’t feel like I had enough time with my rota”.*

### Theme three: the cultural norm – ‘there is a culture change there, for better or for worse’

The majority of doctors now take time out of training after FY2, so many interviewed felt that if they did not, they would be missing out on positive experiences they had seen others take advantage of. This included the opportunity to travel, without risk of losing a training number, with the end of foundation training being seen as a natural point to take a break. ‘You finish one discreet period of training before you start another one, it’s a good time to work abroad or travel’. As part of this participants mentioned the opportunity to increase their income through working locum shifts, though they also mentioned that as a foundation doctor they found it unfair that locum doctors earned more for covering ‘the same job’. Some felt that this would be a good opportunity to locum and save money for their life goals, such as house deposits or paying for travel.

*“I think financially as well, obviously there’s a lot more earning potential. We want to try to ideally save for a deposit for a house and I feel like it’s a lot more feasible with a few months of a good locum rate than foundation rate”.*

After speaking to colleagues and friends, participants mentioned that positive recommendations from those who had experienced an FY3 year was an important influence on their choices. As the majority of trainees now take an FY3 year, the fact that many around them are also considering taking time out is a strong driving force to many:

*“I think it used to be the done thing [going into speciality training] whereas now I think it’s the done thing to not go on and to do something else for a bit”.*

The ability to take control of their time, to use this time to improve their CV in order to be competitive whilst also improving both their work/life balance and their bank balance was seen by many as an opportunity to good to miss:

*“at the end of the day what’s another few years in the grand scheme of life, I’ll be a consultant for forty years at this rate”.*

### Theme four: supporting trainees with the practicalities of their FY3 year – ‘I have absolutely no idea how it works?’

The doctors asked were unsure of governing body and academic requirements that might need to be maintained if they are to take an FY3 year and they felt that there was a lack of support from their foundation programmes with regard to the options available to them. This was combined with worries about returning to training and what the extra requirements might be. They had reservations about how to actually go about taking a year out and how to return successfully.

*“Yeah well I have no idea how that works, I don’t know if I need to get someone re-validate me or if I just can apply come October, I have absolutely no idea how it works”.*

Some of those asked also felt uncertain about the security of their choices with worries about becoming ‘unemployed’ or having no confirmed role within the year, and reservations about negative impact on their pensions or future pay. The general feeling was that the difficulties within the practicalities of securing their FY3 year were outweighed by the ability to ‘get off the treadmill’ and looking after their own resilience and recharging.

It was clear that the foundation doctors asked had strong opinions about their training and were keen to offer suggestions for improvement within FY1 and FY2 as well as supporting FY3s. These are shown in the figure below.

Participants suggested that the support for FY3 doctors should start within foundation training, supporting those applying to specialty training while not neglecting the fact that a majority will be taking a year or more out. This could include support from those who have experience of the positives that have been mentioned but also academic and administrative support to cover common issues such as ‘how to return to training’. Structured FY3 posts are offered within some trusts, including teaching fellow posts, which have strong administrative drive towards supporting doctors achieve their future goals and it may be worth considering using these posts to help model necessary competencies and experiences for those forging their own path.

Participants also suggested that FY3 ‘champions’ might also allow hospitals to structure FY3 programmes which support both the hospital and trainees, having mutual benefit for those involved. This might assist those who feel the uncertainty of locuming is too concerning for them. To allow those doctors to have structured weeks of experience within specialties, particularly those specialties struggling to recruit would also be of mutual benefit for training programmes and doctors taking an FY3 year. Participants also suggested that specialty training could be more flexible in their interview and start dates, allowing some to join throughout the year, and some feeling that flexibility and transparency about time out of training would help.

## Discussion

The reasons that foundation doctors are choosing not to go straight into speciality training centre around the themes of feeling undervalued, career uncertainty and the new cultural norm. They report major feelings of uncertainty regarding career choice at such an early stage of their profession and this challenge was magnified by a perceived lack of flexibility of training and the growing normality of taking time out from training. Trainees feel a lack of support in planning and undertaking an “FY3” year and being helped back into the workforce.

### Comparison to other studies

The central importance of trainee doctors feeling over-worked and under-valued has been raised before in previous work, predominantly from questionnaire surveys [8, 9, 12–14], and is a widespread concern across the NHS workforce [15]. Similarly, a need for more support for Foundation doctors has been recently highlighted by other authors [16]. Our study has highlighted the strength of feeling of current trainees and some of the underlying causes, such as lack of flexibility and poor facilities. Other factors that have been shown to be important to junior doctors in their career decision making are location and a supportive culture [17].

Career uncertainty has been a less often quoted reason for taking time out of training [10] but is unsurprising given the perceived lack of flexibility in postgraduate training. Original career intention at the start of the foundation programme has also been shown to be an important predictor of career intentions at the end of the foundation programme [18]. These ideas that a supportive culture and early experiences are important in determining whether foundation doctors applied for speciality training are supported by further work that showed trainees who felt supported in the early stages of post graduate training were more likely to apply [19]. The level of support and workplace culture was perceived as an even more significant influence into career decision making for female trainees rather than male trainees [20]. A relatively novel theme to emerge from our research is the idea that taking a break from training is becoming the new cultural norm. Trainees view this year as an opportunity to rest and re-charge but also as a necessary step to build a competitive portfolio [10] that will allow them to secure a training post in their desired speciality and location (Table 1).

### Strengths and weaknesses of the study

By using semi structured interviews this study takes a much more in-depth view from an interpretive theoretical stance, to explore the reasons for foundation doctors choosing to take a break from training. Much of the previous research has been descriptive rather than seeking to explain the reasons behind the issue. It is acknowledged that the study involved a relatively small number of the total cohort of junior doctors who were not entering training, albeit from two regions of the UK. However, standard measures of qualitative research were taken to ensure rigour in qualitative work was followed including a clear audit trail, reflexivity and peer coding which reinforce the transferability of the findings.

Finally, the study design was prospective and asked the participants intentions at a given moment in time which may have been subject to change following the interview. Taking an international perspective there is a wide variation in the structure of postgraduate medical education globally. Some countries such as Germany or the Netherlands grant full medical registration on graduation from medical school [21]. While other countries such as the UK, USA, Australia and Canada require a period of ‘internship’ before full registration [21–23]. Though there is significant differences between postgraduate medical education between countries many commonalties exist such as junior doctor wellbeing and changing patient and population needs [21].

### Recommendations

**Fig. 3:**
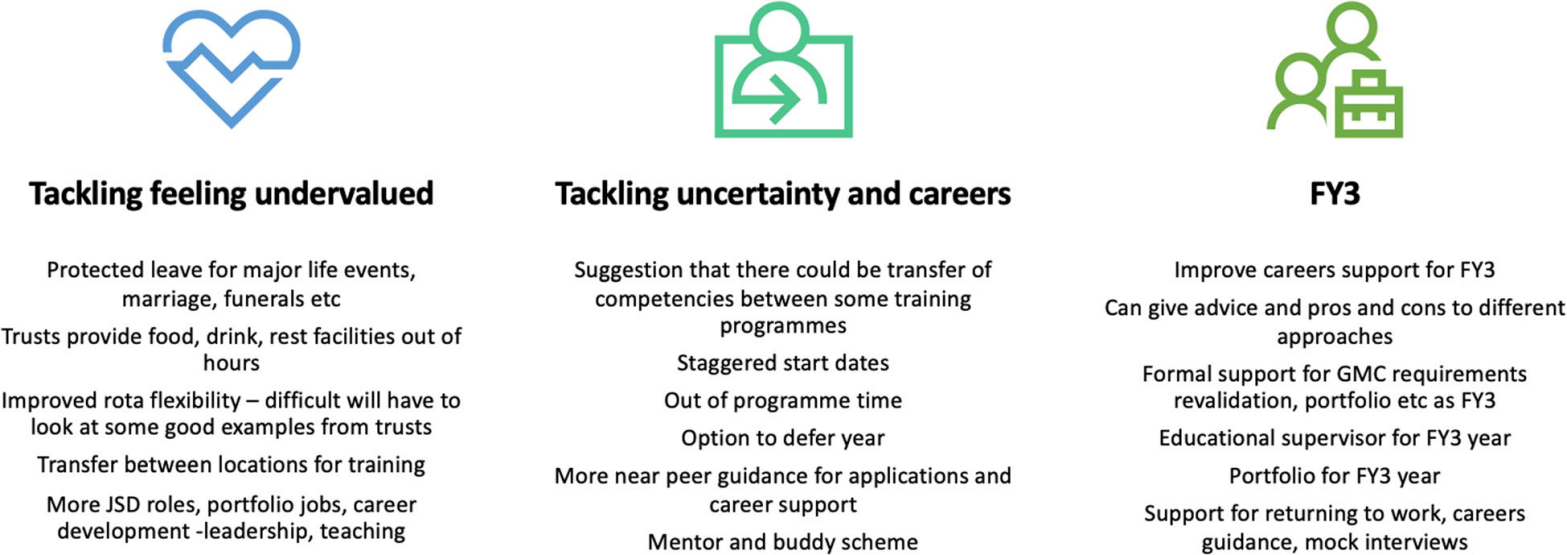
Summarises the suggestions and recommendations put forward by trainee doctors to address the three main themes identified by the interviews

The recommendations offered by trainees to improve training are wide ranging and personal and summarised in Figs. 2 and 3. Although it may be difficult to tailor training pathways to cater for every individual need, nevertheless, training should be more accommodating to allow individuals to develop their personal interests and needs. This may include taking time out of training to pursue personal interests or by building protected time into training that can be used to empower the trainee to re-gain some autonomy back into their life. This sentiment of loss of control and desire for individualised training is supported by previous work in this area [10, 15, 16, 24].

The strong overarching theme for suggestions was support. Can we help doctors and medical students plan their careers to support self-care and achievement of their personal goals? If so we need to embrace the new ‘normal’ and help to maximise its clear benefits.

### Unanswered questions and future research

Future research needs to move past naming the issue and aiming to further understand what is meant by these terms and how organisations can take onboard trainees’ suggestions to improve their working conditions and career development. It is noted that there is ongoing positive work in this area such as the newly agreed junior doctors contract [24] and initiatives such as HEE’s ‘improving doctors working lives’ project [25] and work by the GMC [11]; however these are initial steps rather than a panacea for all workplace related problems. There also needs to be a recognition from both educators and employers that taking a break after foundation training is now a new cultural norm among trainees.

A major area that has been neglected in the ongoing work is addressing and better supporting, what is now the majority of foundation doctors, that are deciding to take a career break. Part of the reason that this has become the norm is that trainees are increasingly searching to regain autonomy and control that they feel is not offered in current training programmes. By not recognising this desire and supporting these trainees at this point in their career we risk further disillusioning them and worsening the vicious cycle of future staff shortages and rota gaps instead of supporting their career and personal development at this crucial stage.

## Conclusion

Taking a year or more out of training after FY2 has become the new cultural norm for UK foundation doctors and reasons for this include feeling undervalued, career uncertainty and the perception that this is now “normal”. Exploring these factors with participants has generated a number of recommendations related to improving the workplace environment, allowing more flexibility in training and supporting those who chose to take an FY3. These results are of interest to workforce planners such as the department of health as any interruption in the conveyer belt of training can have significant workforce implications. It is also potentially of interest to an international audience as it highlights the importance of junior doctor wellbeing and may influence policy to prevent similar cultural norms occurring.

## Data Availability

We are happy to share our raw data and transcripts with the journal.

## Abbreviations

FY1: Foundation year one
FY2: Foundation year two
FY3: Foundation year three
BMA: British Medical Association
GMC: General Medical Council
GP: General Practitioner
GPST3: GP Speciality trainee year three
CCT: Certificate of Completion of Training
HEE: Health Education England
